# Bioprospecting Through Cloning of Whole Natural Product Biosynthetic Gene Clusters

**DOI:** 10.3389/fbioe.2020.00526

**Published:** 2020-06-05

**Authors:** Zhenquan Lin, Jens Nielsen, Zihe Liu

**Affiliations:** ^1^Beijing Advanced Innovation Center for Soft Matter Science and Engineering, College of Life Science and Technology, Beijing University of Chemical Technology, Beijing, China; ^2^Department of Biology and Biological Engineering, Chalmers University of Technology, Gothenburg, Sweden; ^3^Novo Nordisk Foundation Center for Biosustainability, Technical University of Denmark, Lyngby, Denmark; ^4^BioInnovation Institute, Copenhagen, Denmark

**Keywords:** natural product, biosynthetic gene cluster, heterologous expression, sequence-independent cloning, direct cloning

## Abstract

Since the discovery of penicillin, natural products and their derivatives have been a valuable resource for drug discovery. With recent development of genome mining approaches in the post-genome era, a great number of natural product biosynthetic gene clusters (BGCs) have been identified and these can potentially be exploited for the discovery of novel natural products that can find application as pharmaceuticals. Since many BGCs are silent or do not express in native hosts under laboratory conditions, heterologous expression of BGCs in genetically tractable hosts becomes an attractive route to activate these BGCs to discover the corresponding products. Here, we highlight recent achievements in cloning and discovery of natural product biosynthetic pathways via intact BGC capturing, and discuss the prospects of high-throughput and multiplexed cloning of rational-designed gene clusters in the future.

## Introduction

Natural products produced by plant, bacteria, and fungi have served as a crucial source of pharmaceuticals, therapeutic agents and industrially useful compounds, such as antibiotic, antitumor, and anti-infective drugs (Nielsen, 2019). Since the discovery of penicillin in the early 1940s, the identification and bioprospection of natural product biosynthetic gene clusters (BGCs) has attracted much attention (Mullis et al., 2019). With the development of sequencing technologies, the costs of genome sequencing has been reduced, and hereby metagenomics has emerged as a strategic approach to explore unculturable microbes through the sequencing and analysis of environmental DNA. Hereby massive DNA sequence information has become accessible. Moreover, many bioinformatic tools have been developed to uncover putative BGCs, such as antiSMASH 5.0 (Blin et al., 2019), BiG-SCAPE (Navarro-Muñoz et al., 2019), PRISM 3 (Skinnider et al., 2017), MIBiG 2.0 (Kautsar et al., 2019), RODEO (Tietz et al., 2017), and genome-scale metabolic models (Nielsen and Nielsen, 2017). However, there are many technical challenges to translate these putative BGCs into specialized chemicals, resulting in a huge gap in the natural product discover pipeline (Dejong et al., 2016).

Advances in genetics, molecular biology and synthetic biology have been successfully used for natural product discovery (Zhang J. J. et al., 2019; Zhao et al., 2019). It has been estimated that more than 99% of environmental microbes are unculturable under the defined conditions using routine techniques and hard to study using classical experimental approaches (Daniel, 2005). Moreover, a large number of BGCs are not or weakly expressed in native hosts under laboratory conditions, known as ‘silent’ or ‘cryptic’ gene clusters. Thus, besides the traditional screening and characterization methods, such as phenotype screening, insertional mutagenesis, co-culture and elicitor screening (Cacho et al., 2015; Tomm et al., 2019; Zhang X. et al., 2019), cloning and refactoring the putative BGCs in well-defined hosts become attractive approaches for natural product discovery, achieving functional expression of uncharacterized potentially-valuable natural product biosynthetic pathways (Cook and Pfleger, 2019; Xu et al., 2020). While *E. coli*, *Streptomyces*, yeast and *Aspergillus* are often used for heterogeneous expression of BGCs, their applications are still limited by the incompatibility of different transcript regulatory systems and codon preferences among organisms, lack of post-translational protein modifications, insufficient supplies of precursors and co-factors, toxicity of intermediates or final products, and poor assembly of natural products with novel structure (Luo et al., 2016; Nielsen, 2019; Pham et al., 2019). Unlike prokaryotic gene clusters, heterogeneous expression for eukaryotic gene clusters or individual genes introduces additional challenges for heterologous expression, such as intron splicing, insertion of promoters and terminators in upstream and downstream of each coding region, etc. (Alberti et al., 2017; Harvey et al., 2018; Qiao et al., 2019). Many alternative methods have been developed and comprehensively reviewed elsewhere (Baker et al., 2018; Xiong et al., 2019; Deng et al., 2020).

Over the past decade, many approaches have been developed to clone intact BGCs for heterologous expression. However, cloning long genome segments of large gene clusters (range from 20 to ∼200 kb) remains challenging (Fayed et al., 2015). Thus, it’s necessary to develop appropriate vector systems and methods for cloning large-size gene clusters and transfer these genetic segments between different hosts (Liao et al., 2019). In this review, we will focus on recent developments of cloning intact gene clusters from complex genome sequence for natural products discovery, including sequence-independent methods and direct cloning methods, and prospect on high-throughput multiplexed cloning of BGCs.

## Sequence-Independent Methods for Heterogeneous Expression of BGCs

Sequence-independent method constructs expression libraries on sheared genomes from a mixed population (e.g., environmental DNA) or a pure culture, and screens for natural products. Key technologies in sequence-independent methods include high-quality high-molecular-weight DNAs isolation, DNA fragmentation and library construction. This method is particularly useful for scenarios when the genomic information of native hosts is under-characterized. Sequence-independent methods have the advantage to prospect the entire genetic materials, and is possible to cover all the BGCs in the sample and discover novel structural natural products (Zhang J. J. et al., 2019). The approach does, however, require highly efficient screening assays as the library will have a very low fraction of positives. Many groups have successfully used sequence-independent library cloning based on different library construction strategies [e.g., cosmids, fosmids, bacterial artificial chromosomes (BACs), phage artificial chromosomes (PACs)] for natural product discovery (Table 1) (Deng et al., 2017; Nara et al., 2017).

**TABLE 1:** Different strategies for intact natural product BGCs cloning.

Class	Strategies	Principles	Capacity	Advantages	Disadvantages	BGCs
Sequence-independent libraries cloning	Cosmid/fosmid libraries	• Fragmentation, gel-fractionated, ligation and phage packaging	<50 kb	• Not requiring genome sequence data; • Capable of generating natural product with novel structure; • Capable of covering the complete genetic material; • Suitable for cloning environmental DNA.	• Untargeted; • Laborious and time consuming; • Packaging; • Large BGCs maybe spanned into separatebd clones.	Omnipeptin (Libis et al., 2019) Anisomycin (Zheng et al., 2017) Ashimides (Shi et al., 2019) Frigocyclinone (Mo et al., 2019) Locillomycins (Luo et al., 2019)
	PAC/BAC libraries	• Fragmentation, gel-fractionated, and ligation	<300 kb	• Not requiring genome sequence data; • Capable of generating natural product with novel structure; • Capable of covering the complete genetic material.	• Untargeted; • Laborious and time consuming; • Technically challenging for large fragment cloning and DNA extraction.	Atratumycin (Yang et al., 2019) Neoabyssomicin/abyssomicin (Tu et al., 2018) Avermectins (Deng et al., 2017) Murayaquinone (Peng et al., 2018)
	FAC libraries	• Random fragmentation, adaptors ligation, gel-fractionated and ligation	10–200 kb	• Unbiased library; • Not requiring genome sequence data; • Capable of generating natural product with novel structure; • Suitable for fungal BGCs cloning.	• Untargeted; • Laborious and time consuming.	Sesterterpenoid (Clevenger et al., 2017) Benzomalvin A/D (Clevenger et al., 2018) Diketomorpholines (Robey et al., 2018)
Direct cloning	TAR	• *In vivo* homologous recombination of *Saccharomyces cerevisiae*	<100 kb	• Cas9-facilitated high efficiency cloning; • Suitable for cloning large genomic regions.	• Technically challenging to use yeast spheroplasts for highly transformation efficient; • Some false positives; • Requires careful preparation and/or manipulation of gDNA.	Plipastatin (Hu et al., 2018) Scleric acid (Alberti et al., 2019) Brasiliquinones (Herisse et al., 2019).
	LLHR	• RecET-mediated linear-plus-linear homologous recombination in *E. coli*	< ∼52 kb	• Technically easier; • Suitable for cloning small- and mid- BGCs; • Simply for using short recombination homologous arms.	• False positive; • Difficult to clone large-size BGCs; • Require highly specialized capturing vectors; • Multi-rounds selection.	Luminmide A/B (Fu et al., 2012) Bacillomycin (Liu Q. et al., 2016) Streptoketides (Qian et al., 2020)
	ExoCET	• CRISPR/Cas9 digestion, T4 polymerase for *in vitro* annealing and RecET mediated homologous recombination	<∼102 kb	• Technically easier; • Simply for using short recombination homologous arms.	• Low efficiency for clone large-size BGCs; • Require pathway specialized vectors; • False positive.	Salinomycin (Wang et al., 2018) Spinosad (Song et al., 2019)
	CATCH	• Cas9-assisted site-specific cleavage and Gibson assembly	<∼150 kb	• Suitable for cloning large genomic regions.	• Require carefully prepare the target DNA in gel.	Bacillaene (Jiang et al., 2015) Mutanocyclin/SNC1-465 (Hao et al., 2019)
	DiPaC	• Q5 hi-fidelity PCR amplication and Gibson assembly	<22 kb per round	• Technically easier; • Extremely efficient for cloning small- to mid-size BGCs.	• Introduction of new mutations during PCR; • Impractical for large BGCs.	Phenazine fontizine A5 (Greunke et al., 2018) Sodorifen (Duell et al., 2019) Hapalosin (D’Agostino et al., 2018)
	Site-specfic recombinase	• Homologous recombination and circularizing the plasmid *in vivo* or *in vitro* with recombinase	<∼200 kb	• Effectively avoid the introduction of new mutations; • Suitable for high frequency recombination hosts; • Suitable for cloning large BGCs.	• Time-consuming; • Only use in the host with high-frequency natural homologous recombination; • Usually need multi-rounds recombination.	Napsamycin, daptomycin (Du et al., 2015) Siderophore (Hu et al., 2016) Erythromycin (Dai et al., 2015)
	iCatch	• Homologous recombination and *in vitro* self-ligation	<∼20 kb	• Suitable for high frequency recombination hosts.	• Time-consuming; • Only use in the host with high-frequency natural homologous recombination; • Usually need multi-rounds recombination; • Carefully prepare the gnome DNA.	Actinorhodin (Wang et al., 2019)

### Isolation of High-Quality High-Molecular-Weight DNAs

Biosynthetic gene clusters are often 10s of kilobase and even over 100 kb. Thus, methods for preparing high-quality and high-molecular-weight DNAs are critical for successful cloning of intact BGCs. Zhang et al. (2010) reported a method of extracting high-molecular-weight DNAs from a variety of biological materials using CTAB (cetyl trimethyl ammonium bromide) extraction buffer for extraction, followed by phenolchloroform extraction and/or ethanol precipitation. However, this method often causes long DNA molecules shearing, and is used for extracting genomic DNAs up to ∼10 kb. To prepare megabase-size genomic DNA, cellulase and pectinase were firstly used to hydrolyze the cell wall before isolating DNAs from organisms having a cell walls (Zhang et al., 2012). Unlike conventional genomic DNA isolation methods, the protoplast, cells, or the nuclei are embedded in low-melting-point agarose gel matrix to protect large DNA fragments from mechanical shearing during the isolation step (Zhang et al., 2012). Alternatively, for rapid extraction of high-molecular-weight genomic DNA (range from ∼20 to ∼130 kb) from bacteria, plants, and animals, Mayjonade et al. (2016) developed a method that grounds the cell into a fine powder in liquid nitrogen, lyses the cell with SDS-base buffer and finally uses carboxylated magnetic beads to purify the DNA. For more information on the topic of isolating high-quality DNAs, please refer to recent reviews elsewhere (Mohamad Roslan et al., 2017; Green and Sambrook, 2018). Commercial kits for extracting high-molecular-weight DNA are also available (e.g., QIAGEN, Macherey Nagel). A detailed comparison of each method can be found in Supplementary Table S1.

### DNA Fragmentation

Methods available for DNA fragmentation in library construction include enzymatic digestion, sonication, and hydrodynamic shearing (Ignatov et al., 2019). Enzymatic digestion, such as using site-directed restriction enzyme *Sau*3AI to partial digest purified DNA (Clos and Zander-Dinse, 2019), sonication, such as using ultrasound to generate > 120 kb fragments (Bhushan et al., 2011), and hydrodynamic shearing, such as repeatedly passing DNA through a syringe needle (Liu C. et al., 2016), have been widely used for constructions of large-fragment libraries. Compared with enzymatic shearing, sonication and hydrodynamic shearing, which are mechanical fragmentation methods, are more random and enable better control of the size distribution (Li et al., 2017). After fragmentation, DNA samples can be analyzed by fragment analyzer or horizontal agarose gel electrophoresis to test the extent of the yield fragments. Desired size of fragmented DNAs can be separated and extracted using multi-rounds of pulsed field gel electrophoresis (PFGE) with different ramped pulse times (Clos and Zander-Dinse, 2019). Compared to mechanical fragmentation methods, the unevenly distributed restriction sites in the genome may cause inherently biased and incomplete library with enzyme methods.

### Cloning Strategies

After fragmentation and purification, the desired size of fragmented DNAs can be separated by multi-rounds PFGE. The size-selected fragments were end-repaired and ligated to the digested and dephosphorylated vector, such as cosmid, fosmid, BAC, or PAC (Table 1) (Liu et al., 2018; Tu et al., 2018; Clos and Zander-Dinse, 2019). Total ligation products can be transformed into *E. coli* or packaged into a phage for infecting bacteria. The insert size of cosmid/fosmid libraries usually is limited to ∼50 kb, thus, large gene clusters are often split into multiple fragments and reassemble into the whole cluster (Wolpert et al., 2008). Alternatively, PACs can clone inserts ranging in size from 60 to 150 kb, while BACs have a capacity to accommodate and propagate DNA fragments with an average insert size ∼150 kb (Bilyk et al., 2016). Several of these technologies have been turned into products, commercialized by a variety of companies such as Agilent, Bio S&T, and Epicentre Biotechnologies. For unbiased fungal artificial chromosome (FAC) library construction, fragmented DNAs was end-repaired and ligated with *Bst*XI adaptors and after for separating desired sizes DNA by PFGE (Bok et al., 2015). Purified large DNA fragments were ligated into the *Bst*XI-digested shuttle vector-FAC. The average inserts size of FAC libraries was about 150 kb, which can cover most fungal BGCs.

### Successful Applications

The above-mentioned DNA assembly methods were developed in the past decades, and there are already many successful applications (Table 1). For example, Libis et al. (2019) have screened a 10 million cosmid library from soil metagenomic DNA samples using Co-occurrence Network Analysis of Targeted Sequences (CONKAT-seq), and identified omnipeptin. Moreover, Bok et al. (2015) have constructed a novel *Aspergillus*–*E. coli* shuttle FAC expression vector coupling a BAC vector backbone with an autonomous fungal replicating element AMA1 from *Aspergillus nidulans*. Clevenger et al. (2017) had then optimized the FAC-cloning method, and developed fungal artificial chromosomes with metabolomic scoring (FAC-MS) platform for the discovery of fungal specialized metabolites. Utilizing this approach, researchers have screened fragmented genome DNA containing uncharacterized fungal BGCs from *A. terreus*, *A. aculeatus*, and *A. wentii*, and discovered 17 compounds including 15 unreported compounds (Clevenger et al., 2017), including benzomalvin A/D (Clevenger et al., 2018), diketomorpholines (Robey et al., 2018).

In summary, sequence-independent library cloning can generate libraries for both un-sequenced and sequenced DNAs, with each clone harboring 10 to ∼200 kb inserts, promoting the natural product discovery. However, sequence-independent library cloning is usually laborious and time consuming. For example, to reliably cover the whole genome, researchers usually need to generate 10–20 folds genome coverage to obtain the clones harboring BGCs (Bok et al., 2015). This will require optimization of the whole cloning process, for example, the genome extraction should not result in too much genomic fragmentation, the assembly including the transformation step should be highly efficient to generate the required library size, etc. Moreover, desired BGCs may be split into different clones, especially when using cosmid/fosmid libraries to screen and identify large gene clusters.

## Direct Cloning Methods for Heterogeneous Expression of BGCs

**FIGURE 1:**
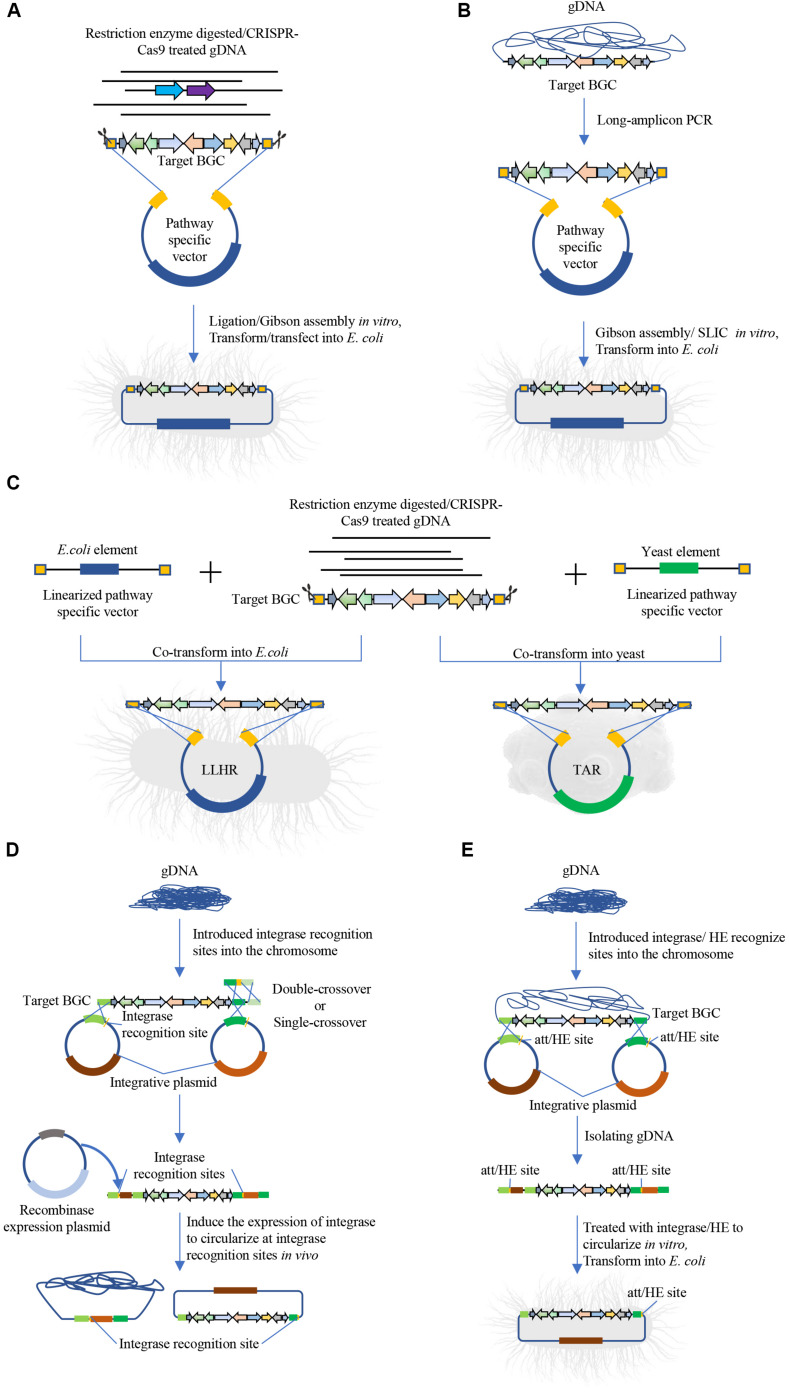
Intact BGC capturing for natural products discovery. **(A)** Direct cloning method based on enzyme digestion and ligation, including ligation or Gibson assembly-based cloning of BGCs, such as CATCH. **(B)** Direct cloning method based on long-amplicon PCR and ligation, such as the DiPaC method. **(C)** Linear–linear homologous recombination (LLHR) mediated by full RecET in *E. coli* or transformation-associated recombination (TAR) in yeast for cloning BGCs. **(D)** Site-direct recombination for cloning BGC *in vivo*, including ϕBT1 integrase-mediated *in vivo* site-specific recombination, Cre/loxP plus BAC. **(E)** Site-direct recombination for cloning BGC *in vitro*, including iCatch, ϕBT1 integrase-mediated *in vitro* recombination.

Direct cloning methods rely on precise bioinformatics to predict BGCs with targeted functions and use specialized cloning method to capture target sequence for expression and/or identification of secondary metabolites. The development of sequencing technologies has resulted in a dramatic reduction of sequencing cost, thus the genome or metagenome information can be easily generated. Meanwhile, several bioinformatic tools have been developed and successfully applied to identify BGCs with potential functions, including PRISM 3 (Skinnider et al., 2017), BiG-SCAPE (Navarro-Muñoz et al., 2019), and antiSMASH (Blin et al., 2019). Direct cloning methods aim to bypass the conventional library generation and screening process and directly isolate gene clusters of interest. Several groups have developed different approaches for direct capture of the BGCs (Figure 1 and Table 1) (Hu et al., 2018; Alberti et al., 2019).

### DNA Isolation and Fragmentation

Methods of DNA isolation and fragmentation for direct cloning are similar with methods used in sequence independent strategies. For DNA fragmentation, physical methods maybe caused target BGCs shearing into fragments. Moreover, the target BGCs are usually too large to find appropriate restriction enzymes that are capable to digest flank homologous regions without also digesting internal targets. To simplify the capture of BGCs, Greunke et al. (2018) developed the direct pathway cloning (DiPaC) method that utilizes long-amplicon PCR to generate target region and Gibson assembly to construct expression plasmids *in vitro*, as shown in Figure 1B. This method is capable of direct cloning small- to mid-sized BGCs (up to < 22 kb per round), resulting in discovery of phenazine fontizine A5 (Greunke et al., 2018), sodorifen (Duell et al., 2019), and heterologous production of anabaenopeptin and erythromycin (Greunke et al., 2018).

The development of advancing genome editing tools, such as clustered regularly interspaced short palindromic repeat–CRISPR-associated protein (CRISPR-Cas) system, has substantially accelerated the process of direct cloning and made it possible to isolate the exact sequence of target BGCs *in vitro* (Lee et al., 2015; Wang et al., 2018; Tao et al., 2019). For example, Jiang et al. (2015) developed Cas9-Assisted Targeting of Chromosome (CATCH) using CRISPR/Cas9 to generate double strand breaks at both ends of target BGCs *in vitro*, and cloned a 78-kb bacillaene gene cluster from *Bacillus subtilis* using Gibson assembly. The concentration of extracted BGCs from target genome or metagenome without enrichment may be too low to yield efficient cloning, and spheroplasts can be used to increase transformation efficiency in transformation-associated recombination (TAR) (Kouprina et al., 2020).

### Cloning Strategies

The development of synthetic biology tools have enhanced cloning of intact BGCs in heterologous hosts (Table 1). Some of these methods are based on exonucleases to “chew back” one of the strands of double-stranded DNAs, thereby exposing complementary single-stranded DNA sequences that can anneal to each other *in vitro* (Figures 1A,B), such as Gibson isothermal assembly (Jiang et al., 2015; Greunke et al., 2018), sequence- and ligation-independent cloning (SLIC) (D’Agostino et al., 2018). Blunt-end ligation have also been emplyed to ligate the CRISPR/Cas9 digested product into a universal vector for λ packaging into phage and transfecting into *E. coli* (Tao et al., 2019) (Figure 1A).

This “chew-back and repair” mechanism has also been applied to clone intact BGCs leveraging on *in vivo* homologous recombination (Figure 1C and Table 1). Several hosts have been widely used for cloning purpose, such as the TAR method in *Saccharomyces cerevisiae* (Kouprina and Larionov, 2019), linear-linear homologous recombination (LLHR) or linear plus circular homologous recombination (LCHR) in *E. coli* (Fu et al., 2012), and exonuclease combined with RecET recombination (ExoCET) (Wang et al., 2018). In these methods, partially digested or randomly sheared DNA was co-transformed into the recombinant host with linearized and pathway-specific vectors containing homology arms that flank the upstream and downstream of the target BGCs. Also, ExoCET can be used to promote homologous recombinations between a linear DNA molecule and a circular plasmid (Fu et al., 2012).

Another type of approach employs site-direct recombination to clone intact BGCs by first integrating specific-vector with the integrase recognition sites in the native host, then the targeted BGCs together with the integrated vector are captured and circularized for heterologous expression (Figures 1D,E and Table 1). This approach requires the native host to have high efficiency of homologous recombination. For example, Dai et al. (2015) intergrated plasmid pEry-up and pEry-down with the BT1 integrase recognition sites *BattP* and *BattB* via single- or double- crossover at both ends of erythromycin BGC, after which genome DNA was carefully isolated and treated with the BT1 integrase to circularize at *att* recombination sequences as a plasmid via *in vitro* site-specific recombination. Similarily, iCatch intergrates homing endonucleases I-SceI and PI-PspI recognition sites flanking the region of interest, after which the genome is isolated and digested with I-SceI or PI-PspI and then self-ligated to clone the target BGC *in vitro* (Wang et al., 2019). Moreover, several groups have developed methods that express recombinases to extract DNA fragments between two integrase recognition sites and circularize the plasmid *in vivo* (Figure 1D), such as phage ϕBT1 integrase-mediated site-specific recombination (Du et al., 2015), Cre/loxP plus BAC (Hu et al., 2016). These plasmids can then be isolated from the native host for heterologous expression.

As shown in Table 1, compared with *in vivo* methods (e.g., TAR, LLHR), *in vitro* cloning methods (e.g., DiPaC, CATCH, iCatch) require carefully preparation of DNA via pre-treatment and purification. Moreover, site-directed recombination methods are suitable for the host with high efficient homologous recombination system. Direct cloning are clearly valuable methods that are well-suited for mining the vast amount of genome for applications in natural product discovery.

### Successful Applications

Over the past decade, direct cloning methods have made great advances and there are already many successful applications (Table 1). For example, Wang et al. (2018) developed a method where exonuclease was combined with ExoCET using the CRISPR/Cas9 cleavage system to digest the target genome and T4 polymerase to pre-anneal linear vector and target DNA before cotransforming into *E. coli*, resulting in cloning of the 106 kb salinomycin cluster and a 79 kb artificial gene cluster (Song et al., 2019). Moreover, TAR has been employed for identification of several novel natural products including orphan cosmomycin (Larson et al., 2017), thiostreptamide S4 (Frattaruolo et al., 2017), and scleric acid (Alberti et al., 2019).

In summary, direct cloning methods can clone intact clusters of interest accurately, and can substantially save time and efforts compared with sequence independent methods. It can also be combined with other modified methods to activated or refactor BGCs in heterologous host. However, current direct cloning methods rely heavily on the quality of genome sequencing and annotation techniques, and have been limited to capture and analyze only one or two clusters each reaction. With the rapidly developed synthetic biology tools available, it will be interesting to see whether these methods can be extended to directly clone all putative BGCs from a give genome in a single reaction.

## Conclusion and Future Perspectives

In the past decades, the developments in synthetic biology, sequencing technology, and bioinformatics have greatly promoted the discovery of BGCs and corresponding products. We can now easily generate vast genome sequences via next-generation sequencing, and annotate them for potential BGCs using defined bioinformatic tools. These predictive BGCs, most of which are putative and do not fall in any known class of BGCs, can be cloned using sequence-independent methods to screen 1000s of clones in one round, or direct cloning methods to clone and analyze targeted BGCs one by one. However, with current technologies it is still challenging to combine the advantages of both methods, and to use direct cloning methods to test all predictive BGCs from an entire genome in a single reaction. Limitations include that high-quality and high-molecular weight DNAs for generating large BGCs is still difficult to isolate, efficient methods for seperating multi-fragments from digested DNA mixture are still missing, highly effective approaches for library construction of targeted BGCs are still limited. The cloning strategies cited in this review will need to be further optimized for BGCs identification and characterization.

In conclusion, with advancements in synthetic biology along with powerful genome mining techniques, we envision a new era of natural product discovery in which BGC cloning will be highly multiplexable, efficient and accurate in a high-throughput manner, leading to the discovery of numerous novel natural products with important biological activities.

## Author Contributions

ZLn, JN, and ZLu drafted the outline and wrote the manuscript. JN and ZLu supervised the research. All authors have read and approved the final manuscript.

## Conflict of Interest

The authors declare that the research was conducted in the absence of any commercial or financial relationships that could be construed as a potential conflict of interest.
