# Supplementary material for: Bioprospecting Through Cloning of Whole Natural Product Biosynthetic Gene Clusters
Source: Front Bioeng Biotechnol. 2020 Jun 5;8:526. doi: 10.3389/fbioe.2020.00526 (PMC7290108; doi:10.3389/fbioe.2020.00526)
Supplement: Supplementary file 1 [file Table_1.doc]

Supplementary Material

# Supplementary Tables

**Supplementary** Table S1 Advantages and disadvantages of different methods for DNA isolation

| Methods | Reagents | Average size | Advantages | Disadvantages | Reference |
| --- | --- | --- | --- | --- | --- |
| Salting-out | Tris-HCl, MgCl_2_, Triton X100, NaCl | ~15 kb | - Cost-effective; - Modified procedures are time effective | - Not suitable for high throughput; - Requires the use of high salt concentrations | (Nasiri et al., 2005; Zhou et al., 2007) |
| CTAB method | Cetyltrimethylammonium bromide (CTAB), EDTA, Tris-HCl, NaCl, proteinase K, phenol/chloroform/isoamyl alcohol, isopropanol, ethanol | 20-300 kb | - Cost-effective; - Modified procedures are time effective - Useful for puriﬁcation of large quantities of polysaccharides samples | - Not suitable for high throughput; - Requires handling and disposal of caustic/toxic organic solvents; - May inhibit downstream enzymatic applications | (Rogers and Bendich, 1994; Thakuria et al., 2009; Grijseels et al., 2016; Werth et al., 2016; Serna-Domínguez et al., 2018) |
| SDS method | Sodium Dodecyl Sulfate (SDS), EDTA, NaCl, proteinase K, hydroxymethyl-hydrochloride, isopropanol, ethanol | 20-300 kb | - Cost-effective; - Modified procedures are time effective | - Precipitation at low temperature; - Poor solubility of SDS may lead to inhibition of enzymes used in subsequent work | (Orsini and Romano-Spica, 2001; Thakuria et al., 2009; Bey et al., 2010; Ouyang et al., 2010; Mayjonade et al., 2016; Werth et al., 2016; Serna-Domínguez et al., 2018) |
| Combined CTAB and SDS method | CTAB, SDS, Tris-HCl, EDTA, NaCl, Proteinase K, phenol/chloroform/isoamyl alcohol, isopropanol, ethanol | 20-300 kb | - Cost-effective; - Modified procedures are time effective and easily for bulk preparations | - Contain abundant polyphenols that interact with DNA or inhibit DNA cloning enzymes | (Möller et al., 1992; Tripathi and Rawal, 1998; Verma et al., 2017) |
| Gel-embedding method | Low melting-point agarose, Tris–HCl, EDTA, lysozyme, 2-mercaptoethanol, proteinase K, N-lauroylsarcosine, lytic enzyme | 25 kb - > 1000 kb,  Chromosome | - Capable of isolation of super high-molecular-weight DNA; - Avoiding mechanical shearing | - Time-cost; - More difficult to prepare and to manipulate during the digestion steps and downstream applications; - Prone to degrade during the isolation process | (Schwartz and Cantor, 1984; Stein et al., 1996; Hume et al., 2001; Ouyang et al., 2010; Zhang et al., 2012; Hao et al., 2019; Wang et al., 2019) |
| Commercial kits | - MagAttract HMW DNA Kit (QIAGEN, Hilden, Germany); - NucleoBond® HMW DNA Kit (Macherey-Nagel, Leicestershire, England); - Wizard® HMW DNA Extraction Kit (Promega, Madison-Wisconsin, USA); - Quick-DNA HMW MagBead Kit (Zymo Research, California, USA) | 10-300kb | - User-friendly; - High throughput; - Less labor-intensive; - High purity; - Environment friendly | - Higher cost than most traditional methods | (Mayjonade et al., 2016; Serna-Domínguez et al., 2018)  <https://www.fishersci.co.uk/gb/en/brands/IFXY1CL7/macherey-nagel.html>  <https://www.zymoresearch.com/products/quick-dna-hmw-magbead-kit> |

**REFERENCES**

Bey, B. S., Fichot, E. B., Dayama, G., Decho, A. W., and Norman, R. S. (2010). Extraction of high molecular weight DNA from microbial mats. *Biotechniques* 49, 631–640. doi:10.2144/000113486.

Grijseels, S., Nielsen, J. C., Randelovic, M., Nielsen, J. C., Nielsen, K. F., Workman, M., et al. (2016). Penicillium arizonense, a new, genome sequenced fungal species, reveals a high chemical diversity in secreted metabolites. *Sci. Rep.* 6, 35112. doi:10.1038/srep35112.

Hao, T., Xie, Z., Wang, M., Liu, L., Zhang, Y., Wang, W., et al. (2019). An anaerobic bacterium host system for heterologous expression of natural product biosynthetic gene clusters. *Nat. Commun.* 10, 3665. doi:10.1038/s41467-019-11673-0.

Hume, M. E., Harvey, R. B., Stanker, L. H., Droleskey, R. E., Poole, T. L., and Zhang, H. B. (2001). Genotypic variation among Arcobacter isolates from a farrow-to-finish swine facility. *J. Food Prot.* 64, 645–651. doi:10.4315/0362-028X-64.5.645.

Marks, P., Garcia, S., Barrio, A. M., Belhocine, K., Bernate, J., Bharadwaj, R., et al. (2019). Resolving the full spectrum of human genome variation using Linked-Reads. *Genome Res.* 29, 635–645. doi:10.1101/gr.234443.118.

Mayjonade, B., Gouzy, J., Donnadieu, C., Pouilly, N., Marande, W., Callot, C., et al. (2016). Extraction of high-molecular-weight genomic DNA for long-read sequencing of single molecules. *Biotechniques* 61, 203–205. doi:10.2144/000114460.

Möller, E. M., Bahnweg, G., Sandermann, H., and Geiger, H. H. (1992). A simple and efficient protocol for isolation of high molecular weight DNA from filamentous fungi, fruit bodies, and infected plant tissues. *Nucleic Acids Res.* 20, 6115–6116. doi:10.1093/nar/20.22.6115.

Nasiri, H., Forouzandeh, M., Rasaee, M. J., and Rahbarizadeh, F. (2005). Modified salting-out method: high-yield, high-quality genomic DNA extraction from whole blood using laundry detergent. *J. Clin. Lab. Anal.* 19, 229–232. doi:10.1002/jcla.20083.

Orsini, M., and Romano-Spica, V. (2001). A microwave-based method for nucleic acid isolation from environmental samples. *Lett. Appl. Microbiol.* 33, 17–20. doi:10.1046/j.1472-765X.2001.00938.x.

Ouyang, Y., Dai, S., Xie, L., Ravi Kumar, M. S., Sun, W., Sun, H., et al. (2010). Isolation of High Molecular Weight DNA from Marine Sponge Bacteria for BAC Library Construction. *Mar. Biotechnol.* 12, 318–325. doi:10.1007/s10126-009-9223-0.

Rogers, S. O., and Bendich, A. J. (1994). Extraction of total cellular DNA from plants, algae and fungi. *Plant Mol. Biol. Man.*, 183–190. doi:10.1007/978-94-011-0511-8_12.

Schwartz, D. C., and Cantor, C. R. (1984). Separation of yeast chromosome-sized DNAs by pulsed field gradient gel electrophoresis. *Cell* 37, 67–75. doi:10.1016/0092-8674(84)90301-5.

Serna-Domínguez, M. G., Andrade-Michel, G. Y., Arredondo-Bernal, H. C., and Gallou, A. (2018). Two efficient methods for isolation of high-quality genomic DNA from entomopathogenic fungi. *J. Microbiol. Methods* 148, 55–63. doi:10.1016/j.mimet.2018.03.012.

Stein, J. L., Marsh, T. L., Wu, K. Y., Shizuya, H., and DeLong, E. F. (1996). Characterization of uncultivated prokaryotes: isolation and analysis of a 40-kilobase-pair genome fragment from a planktonic marine archaeon. *J. Bacteriol.* 178, 591–599. doi:10.1128/JB.178.3.591-599.1996.

Thakuria, D., Schmidt, O., Liliensiek, A. K., Egan, D., and Doohan, F. M. (2009). Field preservation and DNA extraction methods for intestinal microbial diversity analysis in earthworms. *J. Microbiol. Methods* 76, 226–233. doi:10.1016/j.mimet.2008.10.015.

Tripathi, G., and Rawal, S. K. (1998). Simple and efficient protocol for isolation of high molecular weight DNA from Streptomyces aureofaciens. *Biotechnol. Tech.* 12, 629–631. doi:10.1023/A:1008836214495.

Verma, S. K., Singh, H., and Sharma, P. C. (2017). An improved method suitable for isolation of high-quality metagenomic DNA from diverse soils. *3 Biotech* 7, 1–7. doi:10.1007/s13205-017-0847-x.

Wang, J., Wang, J., Lu, A., Liu, J., Huang, W., Cai, Z., et al. (2019). iCatch: a new strategy for capturing large DNA fragments using homing endonucleases. *Acta Biochim. Biophys. Sin. (Shanghai).* 51, 97–103. doi:10.1093/abbs/gmy139.

Werth, S., Reynisdóttir, S., Gudmundsson, H., and Andrésson, Ó. S. (2016). A Fast and Inexpensive High-Throughput Protocol for Isolating High Molecular Weight Genomic DNA from Lichens. *Herzogia* 29, 610. doi:10.13158/heia.29.2.2016.610.

Zhang, M., Zhang, Y., Scheuring, C. F., Wu, C.-C., Dong, J. J., and Zhang, H.-B. (2012). Preparation of megabase-sized DNA from a variety of organisms using the nuclei method for advanced genomics research. *Nat. Protoc.* 7, 467–478. doi:10.1038/nprot.2011.455.

Zhou, X., Li, Q., Zhao, J., Tang, K., Lin, J., and Yin, Y. (2007). Comparison of rapid DNA extraction methods applied to PCR identification of medicinal mushroom Ganoderma spp. *Prep. Biochem. Biotechnol.* 37, 369–380. doi:10.1080/10826060701593282.
